# Rarity and genetic diversity in Indo–Pacific *Acropora* corals

**DOI:** 10.1002/ece3.304

**Published:** 2012-07-06

**Authors:** Zoe T Richards, Madeleine J H Oppen

**Affiliations:** 1 Australian MuseumCollege Street, Sydney, New South Wales, 2010, Australia; 2 Australian Institute of Marine SciencePMB No 3, Townsville MC, Queensland, 4810, Australia; 3 Formerly Australian Research Council Centre of Excellence for Coral Reef Studies, James Cook UniversityTownsville, Queensland, 4814, Australia

**Keywords:** Allelic richness, conservation, heterozygosity, hybridization, microsatellite, population genetics, small population, threatened species

## Abstract

Among various potential consequences of rarity is genetic erosion. Neutral genetic theory predicts that rare species will have lower genetic diversity than common species. To examine the association between genetic diversity and rarity, variation at eight DNA microsatellite markers was documented for 14 *Acropora* species that display different patterns of distribution and abundance in the Indo–Pacific Ocean. Our results show that the relationship between rarity and genetic diversity is not a positive linear association because, contrary to expectations, some rare species are genetically diverse and some populations of common species are genetically depleted. Our data suggest that inbreeding is the most likely mechanism of genetic depletion in both rare and common corals, and that hybridization is the most likely explanation for higher than expected levels of genetic diversity in rare species. A significant hypothesis generated from our study with direct conservation implications is that as a group, *Acropora* corals have lower genetic diversity at neutral microsatellite loci than may be expected from their taxonomic diversity, and this may suggest a heightened susceptibility to environmental change. This hypothesis requires validation based on genetic diversity estimates derived from a large portion of the genome.

## Introduction

As a consequence of their small population size, neutral population genetics theory predicts that rare species will be genetically less diverse than common ones (Kimura 1983). In general, a positive linear relationship is expected between genetic diversity and population size (Wright 1931), whereby as a species expands its population size, there are commensurate increases in genetic diversity. More specifically, for a neutral locus, the expected polymorphism at mutation-drift equilibrium is proportional to the effective population size (*N*_e_ – the number of breeding individuals). Thus, in populations with large *N*_e_, high levels of genetic variation are maintained, and this maximizes adaptive potential (Frankham et al. 2010). Furthermore, the variation in selective pressure between habitats within reefs leads to slightly different local adaptations within a population, and this facilitates higher productivity or stability in the face of disturbance (Palumbi et al. 2008).

The process whereby genetic diversity is lost in small populations is called genetic erosion (Vrijenhoek 1985), and this has been documented in populations of both plants and animals (Nevo et al. 1984; Elstrand and Elam 1993; Baskauf et al. 1994; Frankham 1996). The causal factors of genetic erosion are mostly a combination of strong genetic drift through founder effects or bottlenecks, directional selection, clonality, and/or high levels of inbreeding (Kimura and Ohta 1971; Avise 1994; Willi et al. 2006; Frankham et al. 2010). Genetic erosion is problematic because it tends to reduce the fitness of individuals in a population. Hence, disturbance events, outbreaks of pathogens (Coltman et al. 1999), and other stochastic events can force genetically depleted populations to extinction (Goodman 1987; Elstrand and Elam 1993; Fagen et al. 2002; Frankham et al. 2010).

Under low population size, there is also an elevated risk that favorable alleles may be lost or that deleterious alleles will be fixed and both of these processes diminish the ability of individuals in a population to adapt to, or survive in, changing environments (Lande and Barrowclough 1987). Considering that genetic diversity can have important ecological consequences at the population, community, and ecosystem levels (Hughes et al. 2008), and especially for threatened species (Spielman et al. 2004), it is important that population viability of rare species is examined (Palumbi 2003), and information about genetic diversity is made available for conservation decision making (van Oppen and Gates 2006).

Among the 845 species of zooxanthellate scleractinian coral, published estimates of genetic diversity exist for only 4.6% of species (*n* = 39) (Table 1). These population genetic studies suggest that some high latitude populations of common coral species are vulnerable to genetic erosion (Ayre and Hughes 2004; Underwood et al. 2009), but others are not (Noreen et al. #b^507^). Until now, the level of genetic diversity in rare coral populations has only been examined among species restricted to the Atlantic Ocean (Baums et al. 2005a, 2006, 2010; Foster et al. 2007, 2012; Atchison et al. 2008; Neves et al. 2008; Reyes and Schizas 2010; Palumbi et al. 2012; Goodbody-Gringley et al. 2012); and one species (*Pavona gigantea*) restricted to the far Eastern Pacific Ocean (Saavedra-Sotelo et al. 2011). Thus, the genetic diversity and level of inbreeding in rare Indo–Pacific corals remain to be tested, these being the focus of this study.

**Table 1:** Summary of population genetic data available for zooxanthellate scleractinian corals

Family	Species	Reference
*Pocilloporidae*	*Seriatopora hystrix*	Ayre and Dufty (1994); Ayre and Hughes (2000, 2004); Maier et al. (2005); Underwood et al.(2007); van Oppen et al. (2008); Noreen et al. (#b^507^); Bongaerts et al. (#b^501^); Starger et al. (2010); van Oppen et al. (2011a).
*Pocilloporidae*	*Stylophora pistillata*	Ayre and Hughes (2000); Takabayashi et al.(2003); Ayre and Hughes (2004); Nishikawa (2008)
*Pocilloporidae*	*Pocillopora damicornis*	Stoddart (1984); Benzie et al.(1995); Ayre et al. (1997); Adjeroud and Tsuchiya (1999); Ayre and Hughes (2000); Miller and Ayre (2004, [Bibr b69][Bibr b70]); Ayre and Hughes (2004); Whitaker (2006); Souter et al. (2009); Starger et al. (2010); Combosch and Vollmer (2011); Paz-Garcia et al. (2012).
*Pocilloporidae*	*Pocillopora meandrina*	Magalon et al.(2005)
*Pocilloporidae*	*Pocillopora verrucosa*	Ridgway et al. (2001)
*Acroporidae*	*Isopora cuneata*	Ayre and Hughes (2000, 2004)
*Acroporidae*	*Isopora palifera*	Benzie et al. (1995); Ayre and Hughes (2004)
*Acroporidae*	*Acropora aspera*	Whitaker (2006)
*Acroporidae*	*Acropora austera*	Macdonald et al. (2011)
*Acroporidae*	*Acropora cervicornis*	Vollmer and Palumbi (2006); Baums et al. (2010); Reyes and Schizas (2010).
*Acroporidae*	*Acropora cytherea*	Ayre and Hughes 2004; Márquez et al. (2002); Ladner and Palumbi (2012)
*Acroporidae*	*Acropora digitifera*	Whitaker (#b^510^); Nishikawa (2008); Nakajima et al. (2010)
*Acroporidae*	*Acropora hyacinthus*	Ayre and Hughes (2004); Márquez et al. (2002)
*Acroporidae*	*Acropora millepora*	Ayre and Hughes (2004); Smith-Keune and van Oppen (2006); van Oppen et al. 2011c
*Acroporidae*	*Acropora nasuta*	Mackenzie et al. (2004)
*Acroporidae*	*Acropora palmata*	Baums et al. (2005a, 2006); Reyes and Schizas (2010); Palumbi et al. (2012)
*Acroporidae*	*Acropora tenuis*	Márquez et al. (2002); Underwood et al.(2007); Nishikawa (2008); Underwood (2009)
*Acroporidae*	*Acropora valida*	Ayre and Hughes (2000, 2004)
*Faviidae*	*Plesiastrea versipora*	Rodriguez-Lanetty and Hoegh-Guldberg (2002)
*Faviidae*	*Favia fragum*	Goodbody-Gringley et al. 2010;
*Faviidae*	*Goniastrea aspera*	Nishikawa and Sakai (2003); Nishikawa (2008)
*Faviidae*	*Goniastrea australiensis*	Miller and Ayre (2008b)
*Faviidae*	*Goniastrea favulus*	Miller and Ayre (2008a)
*Faviidae*	*Favia fragum*	Goodbody-Gringley et al. (2010)
*Faviidae*	*Platygyra daedalea*	Miller and Ayre (2008a)
*Faviidae*	*Platygyra sinensis*	Ng and Morton (2003)
*Faviidae*	*Montastrea annularis*	Foster et al. (2007, 2012)
*Faviidae*	*Montastrea cavernosa*	Goodbody-Gringley et al. (2012)
*Faviidae*	*Montastrea faveolata*	Baums et al. (2010)
*Faviidae*	*Diploria strigosa*	Atchison et al. (2008)
*Pectiniidae*	*Mycedium elephantotus*	Yu et al. (1999); Dai et al. (2000)
*Fungiidae*	*Fungia fungites*	Gilmour (2002)
*Fungiidae*	*Heliofungia actiniiformis*	Knittweis et al. (2008)
*Dendrophylliidae*	*Balanophyllia europaea*	Goffredo et al. (2004)
*Siderastreidae*	*Siderastrea stellata*	Neves et al. (2008)
*Siderastreidae*	*Siderastrea radians*	Neves et al. (2008)
*Poritidae*	*Porites lobata*	Polato et al. (Polato et al. (#b^509^)
*Astrocoeniidae*	*Madracis decactis*	Atchison et al. (2008)
*Agariciidae*	*Pavona gigantea*	Saavedra-Sotelo et al. (2011)

*Acropora* (staghorn corals) is the model group for this study because an extensive literature exists on the global ranges of *Acropora* species and we have abundance data that enable us to estimate means global census sizes. *Acropora* are extremely susceptible to coral bleaching, changes in water quality, disease, and predation (Marshall and Baird 2000; Bruno et al. 2007; Pearson 1981). Furthermore, because *Acropora* spp. are particularly important for reef formation, ecosystem function, and biodiversity, and 50% of species in the genus are listed in elevated categories of threat on the IUCN red list (Carpenter et al. 2008), the genetic implications of rarity in *Acropora* have direct conservation significance.

This project is the first to perform a comparative analysis of genetic diversity over a large number of coral taxa. We examine the level of genetic diversity in 14 species of *Acropora* from the Indo–Pacific Ocean (nine rare and five common) encompassing 25 populations from 11 geographic locations to obtain insights into their genetic diversity. We test the null hypothesis that rare species have lower genetic diversity than closely related common congeners, and we generate new a hypotheses pertaining to the susceptibility of *Acropora* corals to environmental change.

## Methods

**Figure 1:**
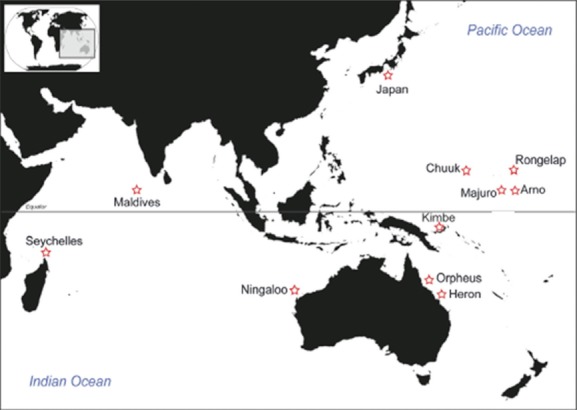
Sampling locations for population genetic analysis.

Samples of 14 species (nine rare, five common – Table 2) were collected from 11 locations across the Indo–Pacific (Fig. 1). Considering that “rarity” can apply not only to patterns of abundance but also to distribution (Brown 1984; Gaston 1994), in this study, we examine the link between genetic diversity and both estimated global census size and maximum global range size.

**Figure 2:**
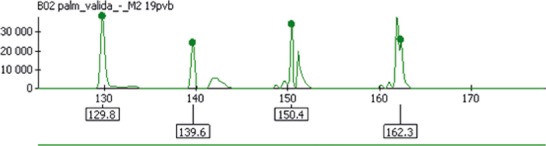
Chromatogram showing multiple peaks in locus Amil2_022 in *Acropora valida* from Orpheus Island. The examples of duplicated alleles that we describe could be evidence of hybridization events; however, duplication was restricted to a single locus (Amil2_022), and does not appear to represent a genome-wide (polyploidization) pattern. Duplication events cannot be explained as scoring errors or PCR artifacts because cloning and sequencing verified genotyping results.

**Table 2:** Summary of species, population sample sizes, and number of loci included in the final analysis

Species	Population	Geographic region	Sample size	Number of loci
*Acropora microphthalma*	Orpheus Island	Central GBR	25	7
	Maldives	North Indian Ocean	12	7
	Seychelles	South Indian Ocean	22	7
	Kimbe Bay	Papua New Guinea	25	7
*A. valida*	Orpheus Island	Central GBR	29	7
	Heron Island	Southern GBR	26	7
	Kimbe Bay	Papua New Guinea	20	7
*A. austera*	Maldives	Indian Ocean	29	6
	Arno Atoll	North Central Pacific	18	5
	Majuro Atoll	North Central Pacific	24	5
	Majuro – 20 branches from single colony	Central Pacific	20	5
*A. millepora*	Ningaloo Reef	Indian Ocean	34	8
	Orpheus Island	Central GBR	27	8
*A. horrida*	Orpheus Island	Central GBR	27	8
A. papillare^*^	Ningaloo Reef	East Indian Ocean	31	7
	Orpheus Island	Central GBR	20	8
	Okinawa – Japan	North Pacific	14	8
A. pichoni^*^	Kimbe Bay	Papua New Guinea	6	7
	Chuuk Lagoon	Central West Pacific	6	7
A. spathulata^*^	Orpheus Island	Central GBR	28	7
A. kirstyae^*^	Orpheus Island	Central GBR	27	8
*A. tortuosa*	Rongelap Atoll	North Central Pacific	12	7
A. jacquelineae^*^	Kimbe Bay	Papua New Guinea	20	7
A. kimbeensis^*^	Kimbe Bay	Papua New Guinea	14	7
A. rongelapensis^*^	Rongelap Atoll	North Central Pacific	12	7
A. walindii^*^	Kimbe Bay	Papua New Guinea	14	8

Species marked with asterisk are rare.

To determine which species have a restricted global distribution, the maximum global range of the 14 species included in this study was quantified using the WorldWide *Acropora* Database, which has 25,000 records based on over 30 years of collections (Wallace 1999). Longitudinal and latitudinal limits for each species were determined from the database, and the range was approximated as elliptical in shape with an area given by: Latitudinal Range/2 × Longitudinal Range/2 × Pi. Species were described as rare if their range is 1/10th or less of the *Acropora* species with the largest global range (*A. valida*). Estimates of global census size were calculated according to Richards et al. (2008). For ease of interpretation, rare species are marked with an asterisk (*) throughout text (e.g., A. pichoni*).

The population sample sizes of the 14 species included in this study range from 6 to 34 individuals (Table 2). It is important to note that conducting population genetic studies on rare species is challenging for a number of reasons, the principal one being that it is exceedingly difficult to obtain sample sizes large enough to warrant interpretations to be made about population-level trends. For corals, the difficulty is further exacerbated by the remote nature of the locations where rare *Acropora* species occur, and the difficulty in identifying rare corals to the species level. Thus, for some of the rare species examined in this study, local populations are so small that it is not feasible to obtain larger population samples. Hence, the small sample sizes and somewhat limited number of species examined prevent a rigorous test of the association between genetic diversity and rarity; however, this study provides a foundation from which the level of genetic diversity in rare corals can be further explored.

All molecular samples examined in this project have matching skeletal voucher specimens that were identified by the author and verified by Dr. Carden Wallace. Small branches (2–5 cm) were collected from individual colonies and stored in absolute ethanol. To minimize sampling across multiple recruitment cohorts and asexually derived clone mates, colony sizes and spacing were standardized (20–50 cm colony size, >20 m between colonies). DNA was extracted from approximately 20 mg of coral branch according to Underwood et al. (2009 – Appendix 1). Precipitated DNA was resuspended in 100 μL 0.1 mol/L Tris pH = 9 and stored at –20°C.

Variation at nine variable tandem repeats (microsatellite markers) was documented using markers previously developed for *Acropora* (Baums et al. 2005b; van Oppen et al. 2007) (Table 3). Microsatellite polymerase chain reaction (PCR) products were initially examined using denaturing gel electrophoresis on the (Corbett GelScan2000, Sydney, Australia). Microsatellite PCR products were visualized using fluorescently labeled forward primers and unlabelled reverse primers. Once it was confirmed via initial GelScan screening that the microsatellites would cross-amplify, genotyping was undertaken following the procedure described below.

**Table 3:** Primer sequences

Locus name	Primer sequence (5′–3′)
Amil2_002	F – ACAAAATAACCCCTTCTACCT
	R – CTTCATCTCTACAGCCGATT
Amil2_006	F – CTTGACCTAAAAAACTGTCGTACAA
	R – GTTATTACTAAAAAGGACGAGAGAATAACTTT
Amil5_028	F – GGTCGAAAAATTGAAAAGTG
	R – ATCACGAGTCCTTTTGACTG
Amil2_022	F – CTGTGGCCTTGTTAGATAGC
	R – AGATTTGTGTTGTCCTGCTT
Amil2_23	F – GCAAGTGTTACTGCATCAAA
	R – TCATGATGCTTTACAGGTGA
Amil2_007	F – TAATGAGCAAACTCATTCATGG
	R – CTTTT CCAAGAGAAGTCAAGAA
Amil2_010	F – CAGCGATTAATATTTTAGAACAGTTTT
	R – CGTATAAACAAATTCCATGGTCTG
Amil2_012	F – TTTTAAAATGTGAAATGCATATGACA
	R – TCACCTGGGTCCCATTTCT

Microsatellites were pooled into three multiplex reactions (Table 4). Each PCR primer was labeled with a different fluorescent dye (TET, HEX, or FAM) and alleles were scored as PCR product size in base pairs. Where more than two bands were observed in an individual, PCR products were cloned for subsequent sequencing to ensure that peaks were true alleles and did not represent nonspecific amplification. Conditions for the PCR included using 150–200 ng of DNA template and 5 μL 2× Qiagen Multiplex PCR kit master mix in a 10 μL reaction in the presence of 1 μL of each primer and 3.25 μL of H_2_O. PCR profile consisted of the initial denaturation step of 15 min followed by 35 cycles of 94° for 30 sec, 50° for 90 sec, and 72° for 60 sec. The mix was incubated at 60°C for 30 min. Three microliters of the PCR product was electrophoresed in a 2% TAE-agarose gel in 1× TAE buffer to assess the yield. Successful products were then cleaned using the Sephadex resin in the Whatman Unifilter 800 system. One microliter of the purified PCR product was transferred to a skirted 96-well plate and sent for genotyping at the JCU Advanced Analytical Centre. Fragment analysis was conducted on the Amersham MegaBase. To minimize genotyping errors, all automated scorings of alleles were checked manually, and rerunning the clean PCR product cleared uncertainties.

**Table 4:** Multiplex reactions

	Locus	Repeat type	Label
Multiplex 1	Amil2_002	(TG)10	HEX
	Ami2_006	(CA)4TA(CA)4	FAM
	Amil5_028	(TCACA)7TCAC(TCACA)4TCACTCACTCACA	TET
Multiplex 2	Amil2_022	(AC)10	TET
	Amil2_23	(AG)7	HEX
Multiplex 3	Amil2_007	(TG)7AG	TET
	Amil2_010	TA(TG)11	FAM
	Amil2_012	GA(CA)6GA(CA)2	HEX

In cases where over two alleles were detected in genotyping, the quality of genotyping results was cross-checked using standard cloning and sequencing techniques. Unlabelled microsatellite PCR products were cloned using the ligation kit, pGEM T easy (Promega, Sydney, Australia) (5 μL ligation buffer, 1 μL pGEM-T Easy Vector, 3 μL PCR product, 1 μL DNA ligase) and incubated for 1–4 h at room temperature or overnight at 4°C. The bacterial cells were transformed with a ligated vector using 60 μL of NM522 competent cells. Cultures were spun in a benchtop centrifuge for 5 min at 4000 rpm. The supernatant was removed and DNA was isolated using the plasmid isolation protocol in the RBC Hyfield Plasmid Mini Kit. The concentration of DNA was determined using a spectrometer and a minimum of 1 μg of purified DNA was dried and sent to Macrogen Inc. (http://www.macrogen.com) for sequencing using SP6 and M13F vector primers.

### Analysis

Microsatellite alleles were scored as a simple function of PCR product size. Genotypes for all loci were manually scored from electrophoretic data. Conformity to the expectations of Hardy–Weinberg equilibrium (HWE) were established using a chi-square test (Miller and Benzie 1997) and significance values were adjusted with Benjamini–Hochberg (BY) correction for multiple comparisons (Benjamini and Yekutieli 2001; Narum 2006) in GenAlex (Peakall and Smouse 2005). Genepop on the web (Raymond and Rousset 1995) was used to test for linkage between loci under the following Markov Chain parameters: 1000 dememorization, 100 batches, and 10,000 iterations per batch. Descriptive statistics, including proportion of polymorphic loci (P), number of alleles per locus (A), and observed and expected heterozygosity, were calculated to illustrate the distribution of genetic diversity within and between populations (Lewis and Zaykin 2001) – Nei's measure was used to correct for uneven sample size in heterozygosity estimates. Allele richness was calculated in Fstat v 2.9.3 (Goudet 2001), and this program was also used to correct for the uneven sample sizes among the populations examined. Allelic diversity and standard genetic distance were computed according to Nei (1987), and significance was corrected for multiple pairwise comparisons (Benjamini and Hochberg 1995).

The extent of inbreeding was summarized by the inbreeding coefficient, *F*_IS_, in Fstat on the Web. This inbreeding coefficient assesses the effects of nonrandom mating within subpopulations, as a measure of reductions in the heterozygosity of individuals. The presence of null alleles (inconsistent amplification of alleles due to mutations in the primer binding region) was assessed in Microchecker v 2.2.3 (van Oosterhout et al. 2004). The probability of identity via sexual reproduction was then examined by calculating the proportion of unique multilocus genotypes (MLGs) at each site (Ng:N) (as per Underwood 2009). In situations where multilocus matches were identified within species, one individual from each pair was removed from subsequent analyses so that each unique genotype was represented only once.

Statistical differences in genetic diversity and level of inbreeding among rare and common species were determined using the Kruskal–Wallace test implemented in SPSS 17 with a single outlier excluded (for further discussion see heterozygosity results for *A. rongelapensis*). The relationships between range size/census size and allelic richness/expected heterozygosity were initially examined with Pearson's Correlation Coefficient in *R,* and regression analysis was used to compare the goodness of fit (*r*^2^). Assumptions of linearity, normality, and homogeneity of variances were assessed through examination of residuals and variables. The significance of linear (*ŷ* = *a* + *b*x) and polynomial relationships (*ŷ* = a + b*x* + c*x*^2) was also examined in multiple regression.

## Results

A total of 531 individuals in 14 species of *Acropora* were genotyped (see Table 2). Thirty-eight percent of initial genotype runs either failed or had multiple peaks, so these samples were genotyped a second time to resolve peaks and 4% were genotyped three times. Overall, 10 species had 100% polymorphic loci (Table 5); however, locus Apam3_166 did not amplify or amplified poorly in all samples, so it was removed from the analysis. Amil2_007 and Amil2_012 also provided mixed results and did not amplify in some populations of some species. For example, Amil2_007 did not amplify A. papillare* from Ningaloo Reef, but did amplify in A. papillare* from Orpheus Island and Japan; Amil2_012 did not amplify in any *A. austera* populations, but worked for all other species examined. Species showing <100% polymorphic loci were: A. papillare*, A. walindii*, *A. valida*, *and A. austera*. In *A. valida*, two populations contained 100% polymorphic loci, but the third (Heron Island) did not (57% of the loci were polymorphic).

**Table 5:** Total number of alleles screened across all loci (*N*), number and percentage of private alleles, number of locus pairs in linkage disequilibrium (LD), percentage polymorphic loci and MLG – identical multilocus genotypes, and probability of identity through sexual reproduction (Ng/N)

Species	Population	*N*	LD	% Polymorphic loci	MLG	Ng/N
*Acropora micropthalma*	Kimbe Bay	51	2	100	0	1
	Seychelles	53	2	100	0	1
	Maldives	35	2	100	0	1
	Orpheus Island	36	2	100	1	1
*A. valida*	Orpheus Island	41	0	100	0	1
	Heron Island	17	0	57.14	3	0.69
	Kimbe Bay	42	0	100	0	1
*A. austera*	Majuro	12	0	50	4	0.83
	Arno	14	0	66.7	0	1
	Maldives	29	0	100	0	1
A. papillare^*^	Ningaloo	41	0	87.5	2	0.93
	Orpheus Island	51	0	100	0	1
	Japan	32	0	87.5	0	1
*A. millepora*	Orpheus Island	48	0	100	0	1
	Ningaloo	62	0	100	2	0.94
A. pichoni^*^	Kimbe Bay	32	0	100	0	1
	Truk	34	0	100	0	1
*A. horrida*	Orpheus Island	43	2	100	0	1
A. jacquelineae^*^	Kmibe Bay	26	0	100	3	0.84
A. kimbeensis^*^	Kimbe Bay	37	1	100	0	1
A. tortuosa^*^	Rongelap Atoll	24	2	100	0	1
A. kirstyae^*^	Orpheus Island	49	3	100	3	0.89
A. spathulata^*^	Orpheus Island	28	0	100	1	0.96
A. walindii^*^	Kimbe Bay	18	0	62.4	0	1
A. rongelapensis^*^	Rongelap Atoll	36	2	100	0	1

### Clonality

The population with the highest degree of clonality was the Majuro *A. austera* population where a single MLG was repeated four times (Table 5) and the probability of asexual reproduction was 17%. The population with the highest occurrence of asexual reproduction was the Heron Island *A. valida* population where Ng/N was 69%. An additional five species displayed evidence of asexual reproduction including the two Ningaloo reef populations (A. papillare* 7% and *A. millepora* 6%), A. jacquelineae* from Kimbe Bay (16%); and A. kirstyae* and A. spathulata* from Orpheus Island (11% and 4%, respectively). The remaining populations of five species were fully sexually produced (*A. horrida*, A. tortuosa*, A. pichoni*, A. kimbeensis*, and A. rongelapensis*).

### Potential polyploidy or multicopy loci

Genotyping showed more than two peaks (three to five) in 15% of the individuals sampled (73 of the 531) (Fig. 2), suggesting either some of the loci are not single-copy in some species or that some species or populations are polyploid. Species showing such patterns included *A. microphthalma*, *A. valida*, *A. austera*, A. kirstyae*, A. kimbeensis*, and A. pichoni*. Cloning and sequencing verified that ≥2 alleles were present for locus Amil2_022 in *A. valida* and A. kimbeensis*, but not for any of the other loci or species. This suggests that this locus has undergone duplication in these species, rather than these species being polyploid. The existence of multiallelic profiles is not accommodated in computer programs that treat codominant markers, so this prevented the inclusion of locus Amil2_022 data in the analysis of the population within which they occur; however, it was included for the other species that showed ≤2 alleles per individual at this locus.

**Figure 3:**
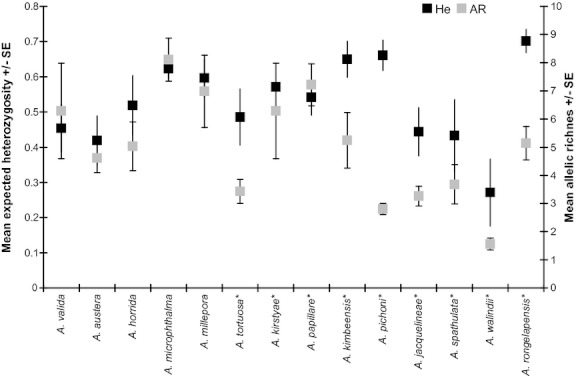
Genetic diversity of rare and common *Acropora* species. Data were pooled for species sampled across multiple populations. Species are listed from most widespread (*A. valida*) to most geographically restricted (A. rongelapensis*). H_e_, expected heterozygosity; AR, allelic richness.

### Heterozygosity

Twenty-nine percent of samples displayed significantly lower observed heterozygosity than expected under HWE at *P* < 0.05. Two common species (*A. millepora* and *A. valida*) have the greatest proportion of loci with significant heterozygote deficits (number of loci in deficit = 62% and 70%, respectively). Significant heterozygote deficits were also detected in rare species (e.g., A. papillare*, A. pichoni*, A. kimbeensis*, and A. spathulata*) and null alleles were encountered 45 times (Appendix 1).

Heterozygote deficits due to null alleles were corrected in 73% of cases. In one case, heterozygote deficit was due to large allele dropout, whereby shorter alleles are preferentially amplified, resulting in the less efficient amplification of large alleles; however, these data were corrected. For the remaining 27% of cases, significant deficits remained after correction for null alleles, suggesting that there are additional reasons for the deficits or there were not enough data to correct the null alleles. Correction reduced *F*_IS_ scores and increased the number of populations with heterozygote excess. For example, all three populations of A. papillare* changed to heterozygote excess after correction for null alleles (note: repeated MLG's were removed before analysis), while the Majuro *A. austera* population remained in deficit after null alleles were corrected. No null alleles were detected in A. rongelapensis* nor A. jacquelineae*. Significant heterozygote excess was detected in A. papillare* at 3 loci and in A. rongelapensis* at 6/7 loci with 100% observed heterozygosity recorded at 3 loci.

Significant genotypic linkage disequilibrium was found in 18 of the performed tests (*P* < 0.05). Among common species, there was no strong link between loci with statistically significant genotypic linkage disequilibrium and populations in HWE disequilibrium. For example, two locus pairs (Amil2_002 and Amil5_002; Amil5_002 and Amil2_010) were linked in all *A. microphthalma* populations; however, only one third of the *A. microphthalma* populations showed significant deviations from HWE (Appendix 1). However, for rare species, loci with statistically significant genotypic linkage disequilibrium also showed significant heterozygote deficits (e.g., A. tortuosa* and A. kirstyae*). The presence of linkage disequilibrium in association with heterozygote deficits may indicate inbreeding or it may be a sign that members of different populations have been sampled (i.e., Wahlund effect).

### Patterns of genetic diversity in rare and common species

**Figure 4:**
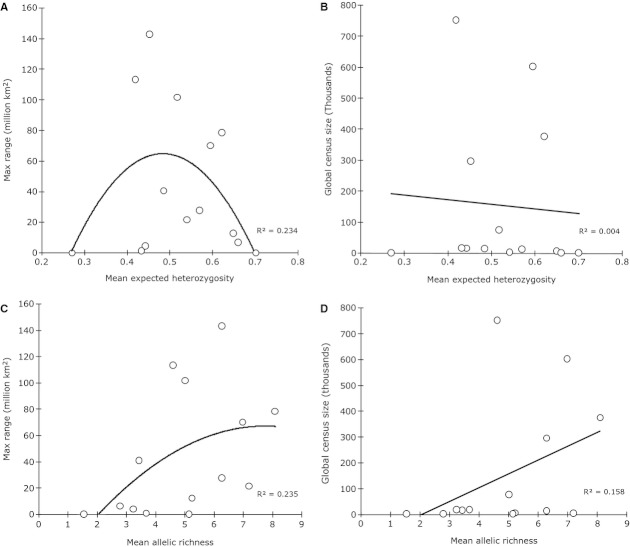
Correlation between range size/estimated global census size and mean expected heterozygosity/allelic richness for 14 species with different distribution/abundance patterns. (A) A nonsignificant nonlinear relationship exists between maximum range size and mean *H*_e_ (*r*^2^ = 0.235, df = 11, *P*-value *x*^2^ = 0.103). (B) A nonsignificant negative linear relationship exists between estimated global census size and mean *H*_e_ (*r*^2^ = 0.005; df = 12; *P* = 0.812). (C) A nonsignificant linear relationship exists between maximum range and allelic richness (*r*^2^ = 0.211, df = 12, *P* = 0.098). (D) A weak nonsignificant positive linear relationship exists between estimated global census size and mean allelic richness (*r*^2^ = 0.159, df = 12, *P* = 0.159).

When both metrics of genetic diversity are plotted together with species ranked from most common to rare, it is obvious that the highest levels of expected heterozygosity occurred in rare species (Fig. 3). This figure also illustrates that among common species, the level of expected heterozygosity and allelic richness was similar; however, the results from these two genetic diversity metrics were quite different for some of the rare species (Fig. 4). For example, in A. pichoni* mean allelic richness was particularly low, while expected heterozygosity was high. Generally patterns of allelic richness and expected heterozygosity at individual loci were extremely variable within species; hence, the large standard error bars.

Acropora walindii* exhibited low allelic richness across all eight loci, suggesting that it is genetically eroded (Table 6). Furthermore, allelic fixation was detected at three loci for A. walindii*. Allele fixation was not restricted to rare species and additional examples of fixed loci are present in the Heron Island *A. valida*, Japan A. papillare*, and Majuro *A. austera* populations (Table 6). The most common locus to be fixed was Amil2_023. Mean allelic richness was greatest in *A. microphthalma* and this is driven largely by the 20 different alleles detected at locus Amil5_028 in the Seychelles population (Appendix 2). Mean expected heterozygosity was greatest in A. rongelapensis*, which was unexpected because it is the rarest of all species examined in this study.

**Table 6:** Patterns of allelic richness between species/populations across the eight loci examined

Species (min. pop. size)	Population	Amil2_02	Amil2_06	Amil5_028	Amil2_022	Amil2_023	Amil2_07	Amil2_010	Amil2_012
Acropora pichoni^*^ (6)	Kimbe Bay	2.879	2.404	3.564	NA	2.24	2	2.182	3.309
	Chuuk	3.309	2.715	3.343	NA	1.745	3	3.233	3.309
	Overall mean	3.12	2.67	3.499	NA	1.965	2.414	2.755	3.208
*A. millepora* (18)	Palm Islands	2.993	3.908	3.948	13.305	3	3	11.224	1.91
	Ningaloo	4.093	4.296	5.791	9.118	5.431	5.937	10.837	6.292
	Overall mean	3.575	4.342	6.074	11.024	4.431	7.263	13.845	5.306
*A. austera* (12)	Majuro	4	4	4	NA	1	NA	1	NA
	Arno	4.714	3.857	3.571	NA	1.999	NA	1	NA
	Maldives	5.659	4.956	2.807	NA	2.994	NA	5.039	NA
	Overall mean	5.772	5.659	4.434	NA	2.899	NA	4.311	NA
A. papillare^*^ (14)	Ningaloo	2	3.382	5.453	7.789	6.228	NA	6.131	4.444
	Palm Islands	5.993	2.7	7.23	11.13	4.378	NA	6.231	3.695
	Japan	6	4	4	4	4	NA	5	1
	Overall mean	6.659	6.307	8.86	10.781	5.476	NA	7.162	5.289
*A. microphthalma* (19)	Kimbe Bay	10.916	4.706	5.598	NA	5.892	5.598	8.921	5.455
	Seychelles	7.696	7.68	11.045	NA	3	6.711	10.909	5.846
	Maldives	3	6	7	NA	4	5	6	6
	Palm Islands	5.711	4.848	3.711	NA	3.968	4.848	5.832	5.711
	Overall mean	10.8	7.086	9.288	NA	5.198	7.554	10.115	6.708
*A. valida* (20)	Palm Islands	4.287	5.597	12.168	PP	3.505	NA	2.69	1.907
	Heron Island	6.808	3	3	PP	1	NA	1.997	1
	Kimbe Bay	6	6	15	PP	4	NA	5	2
	Overall mean	7.022	6.629	13.843	PP	3.608	NA	4.621	2
A. kirstyae^*^ (27)	Palm Islands	2	3.994	6.994	13.65	6.825	5	4.957	5
A. kimbeensis^*^ (14)	Kimbe Bay	6	4	10	PP	3.995	2	6.786	3.929
*A. horrida* (27)	Palm Islands	2.617	7.292	8.347	7.761	3.478	2	3.943	4.833
A. walindii^*^ (14)	Kimbe Bay	1.76	1.791	2.624	2.002	1	1	1.286	1
A. jacquelineae^*^ (19)	Kimbe Bay	3	2.636	3.283	NA	4.895	3	4	2
A. spathulata^*^ (26)	Palm Islands	1.462	2.924	3.993	3.92	2	NA	7.113	4.33
A. rongelapensis^*^ (12)	Rongelap Atoll	6	5	8	NA	3	4	5	5
A. tortuosa^*^ (12)	Rongelap Atoll	4	3	4	4	2	NA	2	5

A wide range of variation in allelic richness was encountered within and between species. Allelic richness could not be calculated for some populations at loci Amil2_022 because more than two alleles were encountered and we hypothesize that locus-specific duplication events have occurred. PP, polyploid; NA, not applicable.

No significant difference (*P* < 0.05) could be detected in the level of expected heterozygosity (*U* = 16, *P* = 0.524) nor allelic richness (*U* = 13.5, *P* = 0.230) between species that are numerically rare versus common. We tested whether there was a significant difference in the level of expected heterozygosity and allelic richness in species that are geographically restricted versus widespread, and again no significant difference (*P* < 0.05) was apparent (*U* = 19, *P* = 0.386 and *U* = 10.5, *P* = 0.109). We further examined the strength of linear and nonlinear relationships between genetic diversity and rarity metrics and the strength of the relationship (Table 7), and found that no significant linear or nonlinear relationships exist between expected heterozygosity and global range size or global census size (Figs. 4a and b). Similarly, there are no significant linear or nonlinear relationships between allelic richness and global range size or global census size (Figs. 4c and d).

**Figure 5:**
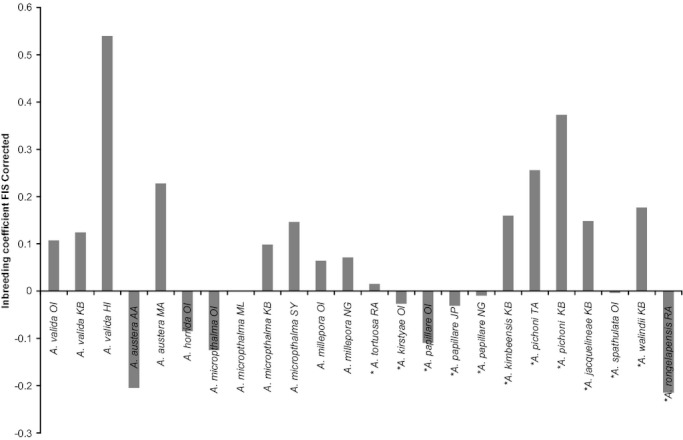
Level of inbreeding (*F*_IS_) in rare and common corals from different locations. Data corrected for null alleles (with the exception of Heron Island *A. valida*; Kimbe Bay and Chuuk Atoll A. pichoni*; and Kimbe Bay A. walindii*). Species are listed from most widespread (*A. valida*) to most geographically restricted (A. rongelapensis*). Rare species are denoted by an asterisk. Positive *F*_IS_ values suggest heterozygote deficit, negative *F*_IS_ values suggest heterozygote excess.

**Table 7:** Regression statistics showing the strength and significance of linear and non-linear relationships between range size or census size and allelic richness or expected heterozygosity

Association	Relationship	*r*^2^	df	*P*	Sig. <0.05
Range size and allelic richness	Linear	0.211	12	0.098	NS
	Polynomial	0.235	11	0.229 (*x* = 0.383; *x*^2^ = 0.570)	NS
Range size and expected heterozygosity	Linear	0.015	12	0.680	NS
	Polynomial	0.235	11	0.229 (*x* = 0.117; *x*^2^ = 0.103)	NS
Census size and allelic richness	Linear	0.159	12	0.159	NS
	Polynomial	0.159	11	0.386 (*x* = 0.809; *x*^2^ = 0.978)	NS
Census size and expected heterozygosity	Linear	0.005	12	0.812	NS
	Polynomial	0.056	11	0.729 (*x* = 0.480; *x*^2^ = 0.457)	NS

NS, nonsignificant.

### Inbreeding

After correction for null alleles, *F*_IS_ values were extremely variable ranging from 0.54 to 0.215. The most highly inbred population was the Heron Island *A. valida* population followed by the Kimbe Bay A. pichoni* population (Fig. 5). There was no significant difference in the level of inbreeding between rare and common species (*U* = 21, *P* = 0.841) or between geographically restricted and widespread species (*U* = 19, *P* = 0.641).

**Figure 6:**
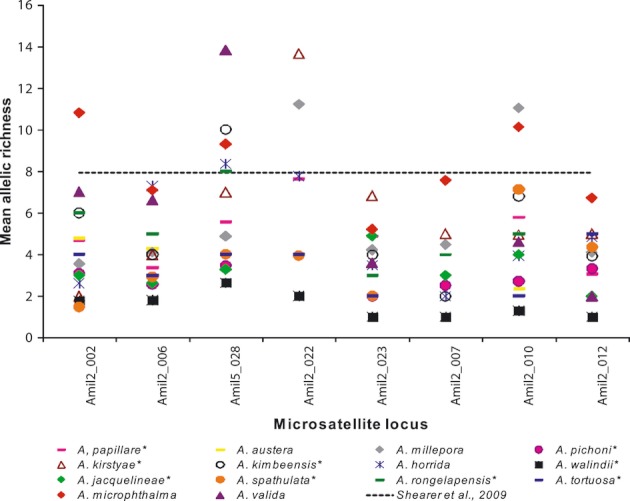
Mean allelic richness for all species/loci examined in this study showing that most *Acropora* species have lower mean allelic richness than what is considered a “conservative mean” in a review of scleractinian coral genetic diversity (Shearer et al. 2009 – pertaining to non-*Acropora* corals only).

## Discussion

Our comparison of genetic diversity shows that, in general, there is a large amount of variability in the level of genetic diversity in *Acropora* spp with varying degrees of rarity. Contrary to expectations of neutral genetic theory, the rare *Acropora* species studied here do not have significantly lower genetic diversity (or higher levels of inbreeding) than common species. Thus, the association between the degree of rarity and genetic diversity in Indo–Pacific *Acropora* is not a simple positive linear relationship. For example, A. walindii* is restricted to Kimbe Bay in Papua New Guinea (Wallace 1999) and it has an estimated global population size of only 1231 ± 615 individuals (Richards et al. 2008). *A. walindii* has exceedingly low genetic diversity and allele fixation is evident at 3 loci. Conversely, A. rongelapensis* has high genetic diversity with heterozygote excess at 6/7 loci and 100% observed heterozygosity (H_O_) at three loci despite having an exceptionally small estimated global census size of only (224 ± 117 individuals) (Richards et al. 2008).

Our results supplement a growing number of rare/common comparative studies in plants that challenge the traditional neutral genetic theory by demonstrating the absence of a significant positive relationship between rarity and genetic diversity (see Karron 1987, 1991; Hamrick and Godt 1990; Young and Brown 1996; Gitzendanner and Soltis 2000; Ellis et al. 2006). Factors which can lead to significant deviations from neutral theory in finite populations have recently been reviewed (Frankham 2012) and include balancing selection, selective sweeps and background selection. While the practical limitations of this study precludes a robust test of neutral genetic theory, our results provide a novel attempt at integrating population demography and genetics, which is urgently needed to address issues related to the effects of climate change on species range and persistence (Lavergne et al. 2010).

The wide range of variation we found between species with differing degrees of rarity undoubtedly reflects a range of population genetic factors. The fixed heterozygosity at three loci in A. rongelapensis*, the rarest species we examined in this study, may be explained in various ways. First, while asexual reproduction could result in fixed heterozygosity, no identical MLGs were identified. Second, high heterozygosity may reflect an old, stable, and persistent population or recruits that are derived from various genetically divergent sources (van Herwerden et al. 2009). However, these explanations are not relevant here as A. rongelapensis* is a member of the large terminal clade in the *Acropora* phylogeny that is considered relatively young (<5 my – Richards et al. 2008) and only a small number of isolated populations of A. rongelapensis* have been located across its continuous distribution range.

The preferred explanation for the finding of 100% observed heterozygosity at 3 loci in A. rongelapensis* is interspecific hybridization. Hybridization has been demonstrated previously in *Acropora* communities, namely in the Caribbean where only three extant species exist, and *A. prolifera* was found to be the product of hybridization between *A. palmata* and *A. cervicornis* (van Oppen et al. 2000; Vollmer and Palumbi 2002). We hypothesize that the individuals of *A. rongelapensis* examined in this study are F1 hybrids; however, the parental lineages have not been established to date. It is likely that hybridization may also have contributed to the higher than expected genetic diversity in other rare species (e.g., A. kimbeensis*). The proposition that some rare species have hybrid ancestries is further supported by a phylogenetic analysis of nuclear and mitochondrial DNA that shows that some rare species are monophylotic for mitochondrial DNA and polyphyletic for nuclear DNA (Richards et al. 2008).

Furthermore, the finding of gene duplication events at Amil2_022 in A. kimbeensis* supports the suggestion that hybridization is a mechanism driving the evolution of genetic diversity in rare species. Single locus duplication events have been reported in another microsatellite study involving *A. millepora* (Wang et al. 2008). Chimerism (where juveniles settle together and fuse – Barki et al. 2002; Puill-Stephan et al. 2009) and the retention of a polar body during fertilization (Baums et al. 2005b) were suggested as possible explanations for the occurrence of more than two alleles per locus. Chimerism is not likely to explain our observations because DNA was examined from a small portion of a single branch only. It is possible that Amil2_022 is not a single copy marker; however, the finding that duplication was restricted to a few individuals indicates that this may also reflect a recent region-specific duplication event caused by transposable element activity, replication slippage, or aberrant crossing over (Bennetzen 2002). Another possible mechanism for tri-allelic patterns is somatic mutations that result in genetic mosaics (van Oppen et al. 2011a). We suggest that gene/genome duplication events should be further examined as mechanisms that drive the evolution of genetic diversity in corals (for further discussion of gene duplication see Stebbins 1940; Ohno 1970; Lynch and Conery 2000; Zhang 2003).

Populations with low genetic diversity (such as A. walindii*, A. pichoni*, and A. jacquelineae* in Kimbe Bay and A. papillare* in Japan) may be more vulnerable and have a higher probability of local extinction following disturbance. Local extinction (the disappearance of a species from part of it range) and ecological extinction (when a species is reduced to such low abundance that, although still present, it no longer plays its typical ecological role) are precursors to global extinction. Data here suggest that some populations of common *Acropora* species may be more vulnerable to local extinction events than previously thought. For example, the *A. valida* population at Heron Island had low genetic diversity, significant heterozygote deficits, and some alleles are fixed. Heterozygote deficits are commonly caused by clonality, null alleles, inadvertently sampling disparate populations (i.e., Wahlund effects, Wahlund 1928) or inbreeding (Wright 1922). In this analysis, clonality and null alleles can be excluded as possible explanations for the observed deficits. Moreover, evidence presented suggests that inbreeding is the primary explanation for heterozygote deficits (e.g., for *A. valida* at Heron Island); however, we cannot exclude the possibility that Wahlund effects may have contributed to the observed deficits.

The concept of corals having low genetic diversity and low *N*_e_ is counterintuitive, given that corals such as *Acropora* have the potential for very high fecundity, in addition to high levels of outbreeding and gene flow (both of which renew genetic variation and increase *N*_e_; Caballero and Hill 1992). A meta-analysis which tested for deviations from the predicted positive linear association between genetic diversity and population size suggests that species with high fecundity (such as fish, oysters, shrimp, and seaweed) have significantly reduced *N*_e_*/N* ratios (Frankham 2012). Thus, despite most corals having high fecundity, they tend to experience very high mortalities in the early life stages, and for any given year, most of the recruited young may be derived from a few large parents, hence there is high variance in progeny number, and consequently, *N*_e_ is likely to be small (Hughes et al. 1992; Hedgecock 1994).

An unexpected outcome of this study was our finding that despite being a taxonomically diverse group in the Indo–Pacific, our overall mean estimate of genetic diversity is quite low in comparison with the mean non-*Acropora* scleractinian diversity estimate reported by Shearer et al. (2009) (7.94 alleles per species specific locus, see Fig. 6). After correction for uneven sample sizes, the mean number of alleles per locus reported across the 25 *Acropora* populations was 4.64 (±0.2 SE) alleles per locus. This level of genetic diversity is just over half of the “conservative mean” presented for non-*Acropora* corals and more closely resembles those reported from isolated high latitude populations (Miller and Ayre [Bibr b69][Bibr b70]). All of the mean estimates of genetic diversity presented here for Indo–Pacific *Acropora* are well short of the number presented for *A. palmata,* a rare species endemic to the Atlantic Ocean (mean 14.4).

To confirm the hypothesis that *Acropora* have lower genetic diversity than other types of corals, genetic diversity estimates could be corrected for differential mutation rates, or genome scans based on, for example, single nucleotide polymorphisms could provide a more accurate picture of functional genetic diversity across a large portion of the genome.

Field observations and mesocosm results in other systems jointly support the hypothesis that genotypic diversity is important in providing different responses to environmental variation (Whitham et al. 2003; Gamfeldt et al. 2005; Hughes and Stachowicz 2009; Wang et al. 2012). However, while our results suggest that some populations of rare and common species have low genetic diversity, it would be erroneous to directly infer anything about the adaptive potential of these populations or the way they will respond to climate change. Microsatellite markers are generally considered neutral (Estoup and Angers 1998) and hence they are not functionally constrained or under selection (Frankham et al. 2010). Thus, for the loci examined here, genetic drift and gene flow, rather than selection, determine their allele number and heterozygosity. Therefore, the finding of low genetic diversity at the relatively small number of neutral loci examined here may not be functionally informative. We recommend that further robust species-specific population estimates at neutral and functional loci are required to fully understand the relationship between genetic diversity, adaptive potential, and persistence.

The conservation implications of this study are found in both theory and practice. Theoretically, we show that there is a large amount of variability in the genetic diversity of *Acropora* corals, which appears to be driven by processes other than rarity. We do not go so far as to suggest that rarity has a minor impact on genetic diversity (see Gillespie 2001; Bazin et al. 2006); however, the finding of nondepletion in some rare species and depletion in some common species suggests that genetic diversity is governed by a complex range of factors. Practically, we emphasize that three of the species which are shown here to have exceptionally low levels of genetic diversity (A. walindii*, A. jacquelineae*, and A. papillare* at Japan) are listed by the IUCN as “Vulnerable,” meaning that they have an elevated risk of extinction risk this century (see Carpenter et al. 2008). The finding of low genetic diversity in these species further exacerbates their threatened status and we propose that targeted species monitoring and management intervention may be necessary to safeguard these species.

## Appendix

**Appendix 1:** Species, population, locus, and number of samples (*N*), alleles (*A*), expected (*H*_e_), and observed (*H*_O_) heterozygosity. Inbreeding coefficient (*F*_IS_), null alleles (Null), corrected inbreeding coefficient (*F*_IS_*C*). Large allele drop-out (LAD), not enough data (NED), not applicable (NA). Asterisks indicate significant deviations from Hardy–Weinberg equilibrium (*P* < 0.05).

		Locus	*N*	*A*	*H*_e_	*H*_O_	*F*_IS_	Null	*F*_IS_*C*
*Acropora microphthalma*	Kimbe Bay	2	23	12	0.849	0.826	0.105	No	0.105
		6	23	5	0.691	0.783	–0.147	No	–0.147
		28	23	6	0.509	0.435	0.096	No	0.096
		22	NA	NA	NA	NA	NA	No	NA
		23	23	6	0.778	0.652	0.136*	No	0.136
		7	22	6	0.478	0.045	0.92*	Yes	*0.119*
		10	25	10	0.728	0.72	0.031	No	0.031
		12	25	6	0.406	0.32	0.232*	No	0.232
	Seychelles	2	22	8	0.747	0.611	0.141	No	0.141
		6	22	7	0.638	0.529	0.191	No	0.191
		28	22	20	0.787	0.389	0.515*	Yes	0.169
		22	NA	NA	NA	NA	NA	NA	NA
		23	22	3	0.575	0.714	–0.171	No	–0.171
		7	22	7	0.702	0.591	0.181*	No	0.181
		10	22	12	0.778	0.545	0.32*	Yes	0.267
		12	22	6	0.635	0.318	0.516*	Yes	0.115
	Maldives	2	19	3	0.661	0.579	0.15	No	0.15
		6	19	6	0.726	0.789	–0.061	No	–0.061
		28	19	7	0.74	0.684	0.102	No	0.068
		22	NA	NA	NA	NA	NA	NA	NA
		23	19	4	0.35	0.421	–0.176	No	–0.176
		7	19	5	0.56	0.333	0.566	Yes	0.186
		10	19	5	0.738	0.533	0.176*	Yes	0.16
		12	19	5	0.616	0.867	–0.397	No	–0.397
	Orpheus Island	2	22	6	0.624	0.773	–0.216	No	–0.216
		6	22	5	0.636	0.818	–0.264	No	–0.264
		28	22	4	0.17	0.091	0.485	Yes	–0.05
		22	NA	NA	NA	NA	NA	NA	NA
		23	22	4	0.458	0.273	0.423*	Yes	0.133
		7	22	5	0.522	0.409	0.238	No	0.238
		10	22	6	0.667	0.864	–0.273	No	–0.273
		12	22	6	0.674	0.909	–0.329*	No	–0.329
*A. valida*	Orpheus Island	2	28	6	0.596	0.357	0.281*	Yes	0.028
		6	25	6	0.728	0.52	0.238*	Yes	0.105
		28	26	13	0.877	0.654	0.231*	Yes	0.225
		22	NA	NA	NA	NA	NA	NA	NA
		23	27	4	0.141	0.074	0.378	Yes	0.043
		7	28	7	0.694	0.179	0*	No	0
		10	28	3	0.364	0.321	0.143	No	0.143
		12	28	2	0.069	0	1*	No	1
	Heron Island	2	17	7	0.78	0.588	0.223	NED	0.223
		6	13	3	0.506	0.077	0.727*	NED	0.727
		28	14	3	0.612	0	0.87*	NED	0.87
		22	NA	NA	NA	NA	NA	NA	NA
		23	16	1	0	0	0	NED	0
		7	NA	NA	NA	NA	NA	NED	NA
		10	17	2	0.111	0.118	–0.045	NED	–0.045
		12	17	1	0	0	0	NED	0
	Kimbe Bay	2	19	6	0.735	0.737	0	No	0
		6	19	6	0.719	0.368	0.535*	Yes	0
		28	19	15	0.904	0.421	0.571*	Yes	0.085
		22	NA	NA	NA	NA	NA	No	NA
		23	19	4	0.389	0.368	0.089	No	0.089
		7	19	4	0.609	0.105	0*	No	0
		10	19	5	0.503	0.368	0.3*	No	0.3
		12	19	2	0.188	0	1*	Yes	0.3
*A. austera*	Majuro	2	12	4	0.462	0.417	0.141	No	0.141
		6	12	4	0.66	0.417	0.405	No	0.405
		28	12	4	0.601	0.25	0.612*	Yes	0.12
		22	NA	NA	NA	NA	NA	NA	NA
		23	11	1	0	0	0	No	0
		7	NA	NA	NA	NA	NA	No	NA
		10	12	1	0	0	0	No	0
		12	NA	NA	NA	NA	NA	NA	NA
	Arno	2	14	5	0.666	0.857	–0.253	No	–0.253
		6	14	4	0.666	0.857	–0.253	No	–0.253
		28	10	4	0.27	0.3	–0.04	No	–0.04
		22	NA	NA	NA	NA	NA	NA	NA
		23	14	2	0.191	0.214	–0.083	No	–0.083
		7	NA	NA	NA	NA	NA	NA	NA
		10	14	1	0	0	0	No	NA
		12	NA	NA	NA	NA	NA	NA	NA
	Maldives	2	28	7	0.733	0.786	–0.054	No	–0.054
		6	28	6	0.625	0.536	0.161	No	0.161
		28	28	3	0.307	0.286	0.087	No	0.087
		22	NA	NA	NA	NA	NA	NA	NA
		23	29	3	0.511	0.586	–0.131	No	–0.131
		7	NA	NA	NA	NA	NA	NA	NA
		10	29	6	0.728	0.655	0.118*	No	0.118
		12	29	6	0.702	0.448	0*	No	NA
*A. papillare*	Ningaloo	2	28	3	0.07	0.036	0.5*	Yes	–0.038
		6	28	4	0.563	0.179	0.692*	Yes	–0.041
		28	28	6	0.735	0.357	0.528*	Yes	0.028
		22	25	9	0.788	1	–0.25*	No	–0.25
		23	25	7	0.626	0.6	0.061	No	0.061
		7	NA	NA	NA	NA	NA	NA	NA
		10	28	7	0.721	0.571	0.225*	Yes	0.015
		12	26	5	0.57	0.462	0.208*	No	0.208
	Orpheus Island	2	20	7	0.639	0.75	0.164	No	0.164
		6	20	3	0.411	0.2	0.532*	Yes	–0.282
		28	20	8	0.8	0.8	0.026	No	0.026
		22	20	14	0.814	1	–0.204	No	–0.204
		23	20	5	0.585	0.15	0.755*	Yes	–0.078
		7	20	3	0.421	0.15	0*	No	0
		10	20	7	0.706	0.75	–0.036	No	–0.036
		12	20	4	0.554	0.8	–0.424	No	–0.424
	Japan	2	14	6	0.755	0.643	0.185*	No	0.185
		6	14	4	0.543	0.571	–0.015	No	–0.015
		28	14	4	0.707	0.071	0.906*	NED	0.906
		22	14	4	0.612	0.857	–0.368	No	–0.368
		23	14	4	0.199	0.143	0.316	No	0.316
		7	14	4	0.487	0.643	0	No	0
		10	14	5	0.691	0.714	0.004	No	0.004
		12	14	1	0	0	0	No	0
*A. millepora*	Orpheus Island	2	26	3	0.556	0.385	0.326	No	0.326
		6	26	4	0.499	0.385	0.248*	No	0.248
		28	26	4	0.362	0.192	0.485*	Yes	0.162
		22	26	16	0.873	0.846	0.05	No	0.05
		23	26	3	0.473	0.308	0.367*	Yes	0.006
		7	18	3	0.642	0	1*	Yes	1
		10	26	13	0.749	0.731	0.044	No	0.044
		12	26	2	0.074	0.077	–0.02	No	–0.02
	Ningaloo	2	32	5	0.409	0.469	–0.131	No	–0.131
		6	32	5	0.445	0.156	0.658*	Yes	0.168
		28	33	6	0.786	0.394	0.51*	Yes	0.155
		22	33	12	0.805	0.727	0.112	No	0.112
		23	33	7	0.597	0.515	0.152	No	0.152
		7	32	7	0.754	0.219	0.718*	Yes	–0.084
		10	33	13	0.865	0.576	0.348*	Yes	0.102
		12	33	7	0.648	0.333	0.498*	Yes	0.022
*A. pichoni*	Kimbe Bay	2	6	6	0.694	0.5	0.362	NED	0.362
		6	6	3	0.611	0	1*	NED	1
		28	6	8	0.847	0.833	0.107	NED	0.107
		22	NA	NA	NA	NA	NA	NED	NA
		23	6	4	0.514	0.5	0.118	NED	0.118
		7	2	2	0.375	0.5	0	NED	0
		10	6	3	0.542	0.167	0.737	NED	0.737
		12	6	6	0.806	0.667	0.259	NED	0.259
	Truk	2	6	6	0.806	1	–0.154	NED	–0.154
		6	6	5	0.667	0.5	0.333	NED	0.333
		28	6	7	0.806	0.833	0.057	NED	0.057
		22	NA	NA	NA	NA	NA	NED	NA
		23	6	2	0.375	0.5	–0.25	NED	–0.25
		7	2	3	0.625	0.5	0.5	NED	0.5
		10	5	5	0.78	0.6	0.333	NED	0.333
		12	6	6	0.806	0.333	0.643*	NED	0.643
*A. horrida*	Orpheus Island	2	26	3	0.144	0.038	0.742*	Yes	0.059
		6	26	8	0.803	0.346	0.582*	Yes	0.019
		28	26	10	0.686	0.346	0.51*	Yes	0.067
		22	22	8	0.834	0.364	0.579*	Yes	0.012
		23	23	4	0.427	0.565	–0.303	No	–0.303
		7	17	2	0.457	0	1*	Yes	–0.333
		10	18	4	0.335	0.389	–0.133	No	–0.133
		12	18	5	0.466	0.611	–0.285	No	–0.285
*A. jacquelineae*	Orpheus Island	2	17	3	0.631	0.529	0.191	No	0.191
		6	17	3	0.258	0.176	0.342	No	0.342
		28	17	4	0.306	0.176	0.448	No	0.448
		22	NA	NA	NA	NA	NA	No	NA
		23	19	7	0.578	0.789	–0.343	No	–0.343
		7	11	3	0.657	0.545	0.216*	No	0.216
		10	11	4	0.442	0.455	0.02	No	0.02
		12	11	2	0.236	0.091	0.643	No	0.643
*A. kimbeensis*	Kimbe Bay	2	13	6	0.719	0.385	0.496*	Yes	0.163
		6	13	4	0.666	0.538	0.229	No	0.229
		28	13	10	0.858	0.615	0.319	Yes	0.319
		22	NA	NA	NA	NA	NA	No	NA
		23	14	4	0.459	0.143	0.708*	Yes	0.071
		7	13	2	0.497	0.462	0.111	No	0.111
		10	14	7	0.694	0.857	–0.2	No	–0.2
		12	14	4	0.656	0.429	0.378	No	0.378
*A. tortuosa*	Rongelap Atoll	2	12	4	0.462	0.25	0.492*	Yes	0.135
		6	12	3	0.559	0.25	0.582*	Yes	–0.021
		28	12	4	0.608	0.25	0.616*	LAD	0.616
		22	12	4	0.563	0.917	–0.603	No	–0.603
		23	12	2	0.153	0.167	–0.048	No	–0.048
		7	NA	NA	NA	NA	NA	NA	NA
		10	12	2	0.278	0.333	–0.158	No	–0.158
		12	12	5	0.778	0.833	–0.028	No	–0.028
*A. kirstyae*	Orpheus Island	2	24	2	0.499	0.125	0.759*	Yes	–0.004
		6	24	4	0.582	0.167	0.724*	Yes	–0.129
		28	24	7	0.777	0.583	0.269	LAD	0.269
		22	23	14	0.765	0.913	–0.173	No	–0.173
		23	23	7	0.433	0.348	0.218	No	0.218
		7	22	5	0.249	0.136	0.471*	Yes	–0.122
		10	23	5	0.631	0.826	–0.288	No	–0.288
		12	23	5	0.632	0.609	0.06*	No	0.06
*A. spathulata*	Orpheus Island	2	26	2	0.038	0.038	0*	No	0
		6	26	3	0.443	0.077	0.832*	Yes	–0.098
		28	24	4	0.666	0.5	0.269*	No	0.269
		22	13	4	0.388	0.154	0.628*	Yes	0.13
		23	12	2	0.153	0.167	–0.048	No	–0.048
		7	NA	NA	NA	NA	NA	No	NA
		10	20	8	0.8	0.9	–0.1	No	–0.1
		12	22	5	0.546	0.636	–0.142	No	–0.142
*A. walindii*	Kimbe Bay	2	14	2	0.408	0.571	–0.368	NED	–0.368
		6	13	2	0.426	0.154	0.662	NED	0.662
		28	12	5	0.681	0.5	0.305	NED	0.305
		22	14	3	0.518	0.5	0.071	NED	0.071
		23	14	1	0	0	0	NED	0
		7	2	1	0	0	0	NED	0
		10	14	3	0.135	0.143	–0.02	NED	–0.02
		12	14	1	0	0	0	NED	0
*A. rongelapensis*	Rongelap Atoll	2	12	6	0.722	1	–0.347*	No	–0.347
		6	12	5	0.726	0.75	0.01	No	0.01
		28	12	8	0.83	0.833	0.039*	No	0.039
		22	NA	NA	NA	NA	NA	No	NA
		23	12	3	0.538	1	–0.846*	No	–0.846
		7	12	4	0.726	0.917	–0.222*	No	–0.222
		10	12	5	0.694	0.667	0.083	No	0.083
		12	12	5	0.674	1	–0.451	No	–0.451

## Appendix

**Appendix 2:** Number of alleles per locus

Species	Amil2_02	Amil2_06	Amil5_028	Amil2_022	Amil2_023	Amil2_07	Amil2_010	Amil2_012
*Acropora micropthalma KB*	12	5	6	0	6	6	10	6
*A. micropthalma SY*	8	7	20	0	3	7	12	6
*A. micropthalma ML*	3	6	7	0	4	5	5	5
*A. micropthalma PI*	6	5	4	0	4	5	6	6
*A. valida PI*	6	6	13	0	4	7	3	2
*A. valida HI*	7	3	3	0	1	0	2	1
*A. valida KB*	6	6	15	0	4	4	5	2
*A. austera MA*	4	4	4	0	1	0	1	0
*A. austera AA*	5	4	4	0	2	0	1	0
*A. austera ML*	7	6	3	0	3	0	6	6
*A. millepora PI*	3	4	4	16	3	3	13	2
*A. millepora NG*	5	5	6	12	7	7	13	7
*A. horrida PI*	3	8	10	8	4	2	4	5
*A. papillare* NG*	3	4	6	9	7	0	7	5
*A. papillare* PI*	7	3	8	14	5	3	7	4
*A. papillare* JP*	6	4	4	4	4	4	5	1
*A. pichoni* KB*	6	3	8	0	4	2	3	6
*A. pichoni* L*	6	5	7	0	2	3	5	6
*A. jacquelineae* KB*	3	3	4	0	7	3	4	2
*A. kimbeensis* KB*	6	4	10	0	4	2	7	4
*A. tortuosa* RA*	4	3	4	4	2	0	2	5
*A. kirstyae* PI*	2	4	7	14	7	5	5	5
*A. spathulata* PI*	2	3	4	4	2	0	8	5
*A. walindii* KB*	2	2	5	3	1	1	3	1
*A. rongelapensis*RA*	6	5	8	0	3	4	5	5
